# Direct Synthesis of cubic shaped Ag_2_S on Ni mesh as Binder-free Electrodes for Energy Storage Applications

**DOI:** 10.1038/s41598-019-46583-0

**Published:** 2019-07-12

**Authors:** Arunachalam Arulraj, N. Ilayaraja, V. Rajeshkumar, M. Ramesh

**Affiliations:** 1 Department of Physics, University College of Engineering – Bharathidasan Institute of Technology (BIT) campus, Anna University, Tiruchirappalli, India; 2 Functional Materials Division, CSIR- Central Electrochemical Research Institute (CECRI), Karaikudi, India; 3 Department of Chemistry, National Institute of Technology, Tiruchirappalli, India

**Keywords:** Energy science and technology, Nanoscience and technology

## Abstract

A facile approach of chemical bath deposition was proposed to fabricate direct synthesis of silver sulphide (Ag_2_S) on nickel (Ni) mesh without involvement for binders for supercapacitor electrodes. The phase purity, structure, composition, morphology, microstructure of the as-fabricated Ag_2_S electrode was validated from its corresponding comprehensive characterization tools. The electrochemical characteristics of the Ag_2_S electrodes were evaluated by recording the electrochemical measurements such as cyclic voltammetry and charge/discharge profile in a three electrode configuration system. Ag_2_S employed as working electrode demonstrates notable faradaic behaviour including high reversible specific capacitance value of 179 C/g at a constant charge/discharge current density of 1 A/g with high cyclic stability which is relatively good as compared with other sulphide based materials. The experimental results ensure fabricated binder-free Ag_2_S electrodes exhibits better electrochemical performance and suitable for potential electrodes in electrochemical energy storage applications.

## Introduction

In the past few decades, considerable research efforts have been focused on alternative energy storage and conversion devices with significant efficiency with low cost such as batteries, solar cells, fuel cells, electrochemical capacitors etc^1–3^. Among these, electrochemical capacitors commonly known as supercapacitors, have been known for promising energy storage devices owing to its uniqueness such as high power density, outstanding reversibility, rapid recharge ability, extended life cycle and low cost fabrication^4,5^. These properties have drawn much attention as energy devices in addition to that of batteries. Even though, supercapacitors have smaller energy densities than batteries, it has a potential of delivering high power density owing to its rapid ion exchange process^6^. The mechanism of energy storage in supercapacitors is inherently rapid because it involves simple ions movement to and fro on the electrode surfaces. Based on the charge storage mechanism, it is broadly classified into two type viz.: (i) Pseudocapacitors (PSCs) and (ii) Electrical double layer capacitors (EDLCs). Currently, EDLCs exhibit higher power density, but it suffers from lower energy density, while PSCs possess a high specific capacitance value of 10–100 times higher than that of EDLCs^7–9^.

Recently, layered type transition metal chalcogenides (TMC), metal carbides and metal nitrides are demonstrated as elevated staging supercapacitive materials^10–13^. Similarly, other transition metal chalcogenides such as NiS, CoS, CuS and ternary metal sulphides are explored as better pesudocapacitive materials^14,15^. The sulphide based materials exhibits excellent electronic, physio-chemical and good conductivity properties, which can serves as an alternative electrode for energy storage applications. Ag_2_S belonging to I–VI compound semiconductor with the band gap of 1–2 eV is one of the promising material for energy storage and conversion devices. Owing to its photo-electric, bandgap and thermal properties it finds own path in different applications such as IR detectors, photoconductors etc^16–18^. In general, fabrication of metal chalcogenides thin films can be deposited on different substrates using various physicochemical techniques like Chemical Vapour Deposition, SILAR, Electrodeposition and so on. Among the other techniques, Chemical Bath Deposition (CBD) in aqueous medium is one the simplest and most economical route to prepare Ag_2_S thin films. CBD, technique has more advantages like low temperature aqueous method for depositing large-area thin films of semiconductors with good uniformity and better adhesion than the others. Besides, it does not require any vacuum system or sophisticated instrument and also the starting materials used in this present work are commonly available and much cheap. Dhumere *et al*. studied the effect of bath temperature and addition of complexing agent on deposition of Ag_2_S thin films using CBD method^19^. Followed by Dhumere, Grazdanov *et al*. reported the different metal sulphide and selenides thin film using electroless deposition method. They studied the optical and electrical properties of Ag_2_S thin films^20^. Mo.*et al*. reported Ag_2_S with graphene nano composites for supercapacitor applications using hydrothermal synthesis^21^.

In this work, we have demonstrated direct growth of Ag_2_S on the surface of Nickel (Ni) mesh using CBD technique at a low temperature of 6 °C without addition of any complexing agents like EDTA, TEA or citric acid and binders. Then the prepared Ag_2_S materials on Ni mesh were employed directly without any further process for electrochemical characterization. From the electrochemical studies, it observed that the Ag_2_S materials showed better performance for energy storage applications.

## Results and Discussion

### Morphological and elemental analysis

**Figure 1:**
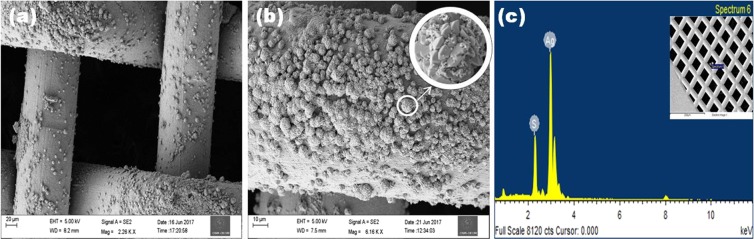
FESEM images of (**a**) Ag_2_S deposited on Ni mesh, (**b**) magnified image and (**c**) EDAX spectrum of Ag_2_S.

Electrochemical performance of the materials are depends on its morphology. Therefore, the morphology of the binder-free Ag_2_S on Ni mesh is observed from the FE-SEM analysis and the micrographs with different magnifications are presented in Fig. 1. The deposition of Ag_2_S over the surface of Ni mesh is shown in Fig. 1a and the individual particles attached on the Ni mesh are clearly observed in Fig. 1b. The particles are truncated cubic in structure which agglomerated to form ball like structures and Fig. 1c shows the elemental composition present in the sample using EDAX analysis, it shows the atomic weight percentage of Ag and S was found to be 67.50 and 32.50% which reveals the Ag_2_S formation.

### Phase structure and functional analysis

**Figure 2:**
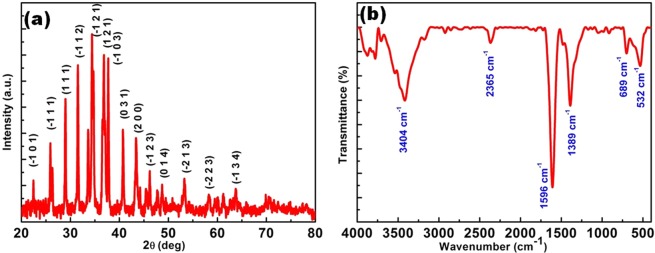
(**a**) XRD pattern and (**b**) FT-IR spectrum of chemical bath synthesized Ag_2_S.

To identify the crystalline behaviour as well as phase structure of the synthesized Ag_2_S, X-Ray diffraction analysis has been carried out and the obtained diffraction patterns are presented in Fig. 2a. The XRD pattern of the prepared sample shows sharp diffraction pattern indicating that Ag_2_S is well crystalline in nature. The observed 2θ values of intense diffraction are 22.38, 25.98, 28.92, 31.55, 34.49, 36.79, 37.71, 40.7, 43.45, 46.28, 53.27° corresponds to lattice planes (−1 0 1), (−1 1 1), (1 1 1), (−1 1 2, (−1 2 1), (1 2 1), (−1 0 3), (0 3 1), (2 0 0), (−1 2 3) and (−2 1 3) of Ag_2_S respectively. The measured diffraction angles and interplanar spacing are in close agreement with the standard diffraction pattern of acanthite phase of Ag_2_S (JCPDS No.: 01-014-0072). The average crystallite size of the as-synthesized Ag_2_S is calculated using Debye Scherer relation and is found to be around 48–86 nm^22^. Mostly, the synthesis of sulphides based materials by chemical approaches results in formation of mixed phase structure because of its complex stoichiometric nature^23^. But in this present case, there is no formation of additional peaks confirming the phase of the synthesized materials.

In order to understand the nature of functional groups present in the synthesized Ag_2_S, FT-IR analysis is carried out and the spectra is given in Fig. 2b. The sharp band appears in the region of 1596 cm^−1^, 1389 cm^−1^ and broad band around 3400 cm^−1^. The broad band at the region of 3400 cm^−1^ is due to the presence of adsorbed water molecules^24^, and the other bands observed at 1596, 1389, and 532 cm^−1^ corresponds to C = S stretching, N-C-N symmetric stretching and N-C-S bending vibrations respectively^25,26^. A peak with relatively weak intensity observed at 665 cm^−1^ corresponds to the sulphur-sulphur bond in metal sulphides^27^.

### Chemical composition analysis

**Figure 3:**
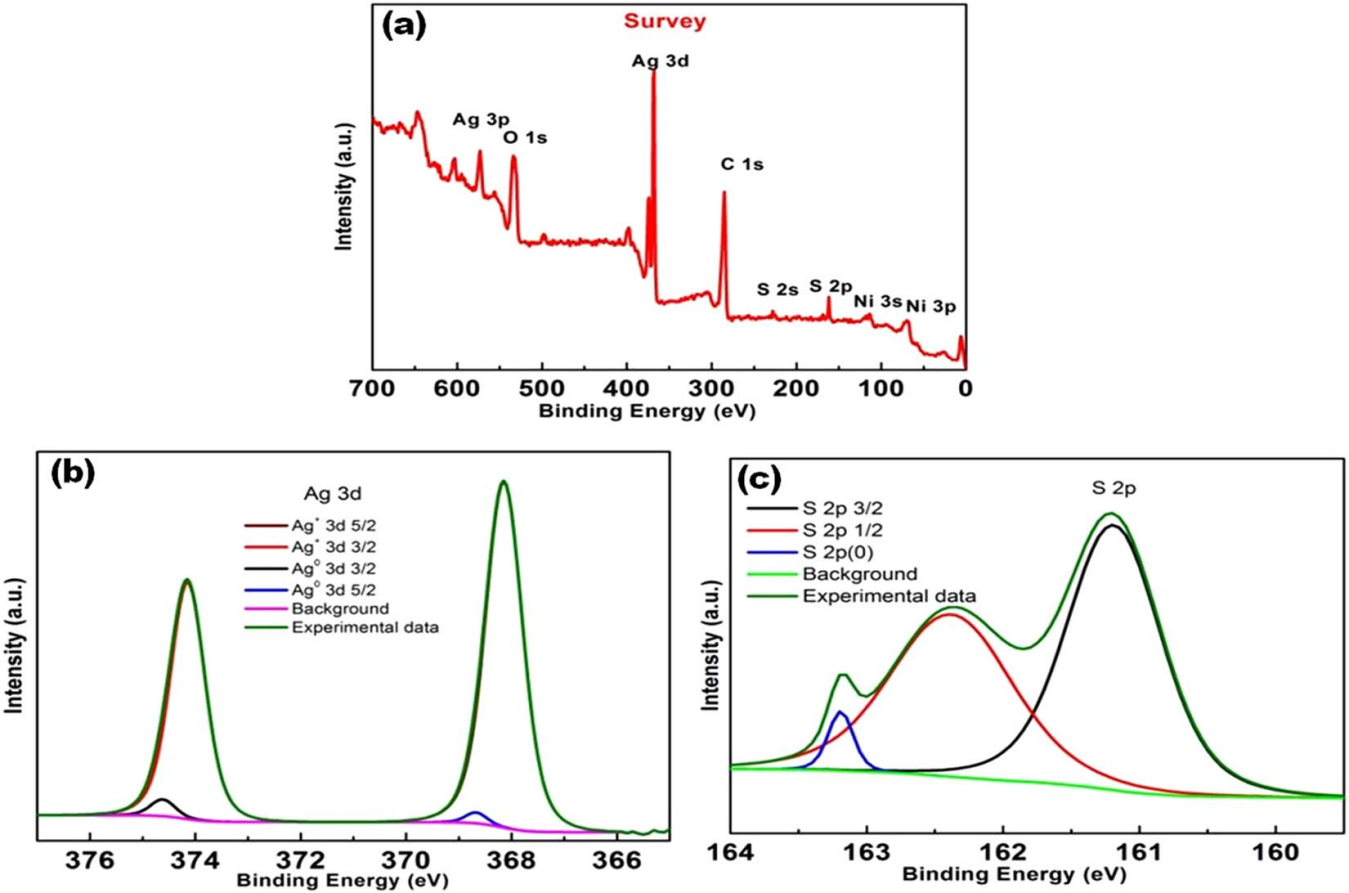
XPS spectrum of Ag_2_S nanoparticles (**a**) survey spectrum (**b**,**c**) High resolutionspectra of Ag and S.

XPS analysis reveals the information about the chemical state and the elemental composition of the synthesized Ag_2_S materials. An extensive scan of survey spectrum of Ag_2_S (Fig. 3a) with the physically powerful existence of Ag 3d (365.6 eV, 372.7 eV) and S 2p (16.21 eV, 163.6 eV) doublets along with C 1 s (284.3 eV), O 1 s (534.3 eV) and other Ag- and S- related core-level binding energy and auger peaks. The apparent existence of Ag 3d and S 2p doublets indicates the formation of Ag_2_S nanoparticles. High resolution scan of Ag 3d and S 2p core-level spectra are shown in Fig. 3b,c respectively. Ag 3d binding energy spectrum is deconvoluted and fitted with two silver doublets. Binding energy centred at 374.1 eV (3d_3/2_) and 368.1 eV (3d_5/2_) contribute to Ag^+^ silver sulphide formation, whereas peaks at 374.6 eV (3d_3/2_) and 368.6 eV (3d_5/2_) are endorsed to Ag°, metallic state of silver in the metal sulphide nanoparticles. A relative shift of about 0.5 eV is observed for the Ag° oxidation state towards high energy as compared to Ag^+^ state. All these experimental findings are much in streak with existing values of Ag_2_S^28–30^.

Related to the Ag 3d, a high-resolution binding energy spectrum of S 2p has been observed (Fig. 3c). The recorded S 2p binding energy spectrum is deconvoluted for spin orbit splitting of metal sulphide S^2+^, centred on 161.2 eV (2p_3/2_) and 162.3 eV (2p_1/2_). A spin orbit splitting with an intensity ratio of 0.52 (expected theoretical value is 0.5) for S 2p matches with an earlier reported values and suggest the formation of Ag_2_S^28,30,31^.

### Electrochemical measurements

**Figure 4:**
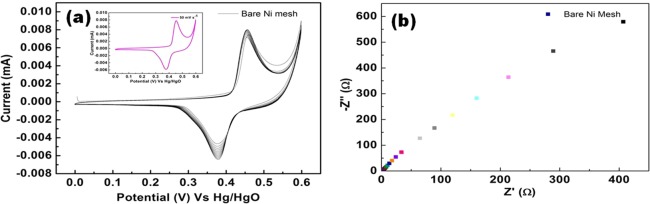
Electrochemical analysis of bare Ni mesh.

Electrochemical characteristics of fabricated binder-free Ag_2_S working electrode are evaluated using cyclic voltammetry (CV), galvanostatic charge/discharge (GCD) and electrochemical impedance spectroscopy (EIS) with a conventional three electrode system. Initially, the CV profile of Ag_2_S electrodes is recorded in 20% KOH electrolyte solution with different scan rates of 5–50 mV s^−1^ and the potential window of 0–0.6 V respectively. Before evaluating the electrochemical performance of the Ag_2_S deposited over the Ni mesh, the performance of bare Ni mesh has been done for comparative purposes and it is represented as Fig. 4a,b.

**Figure 5:**
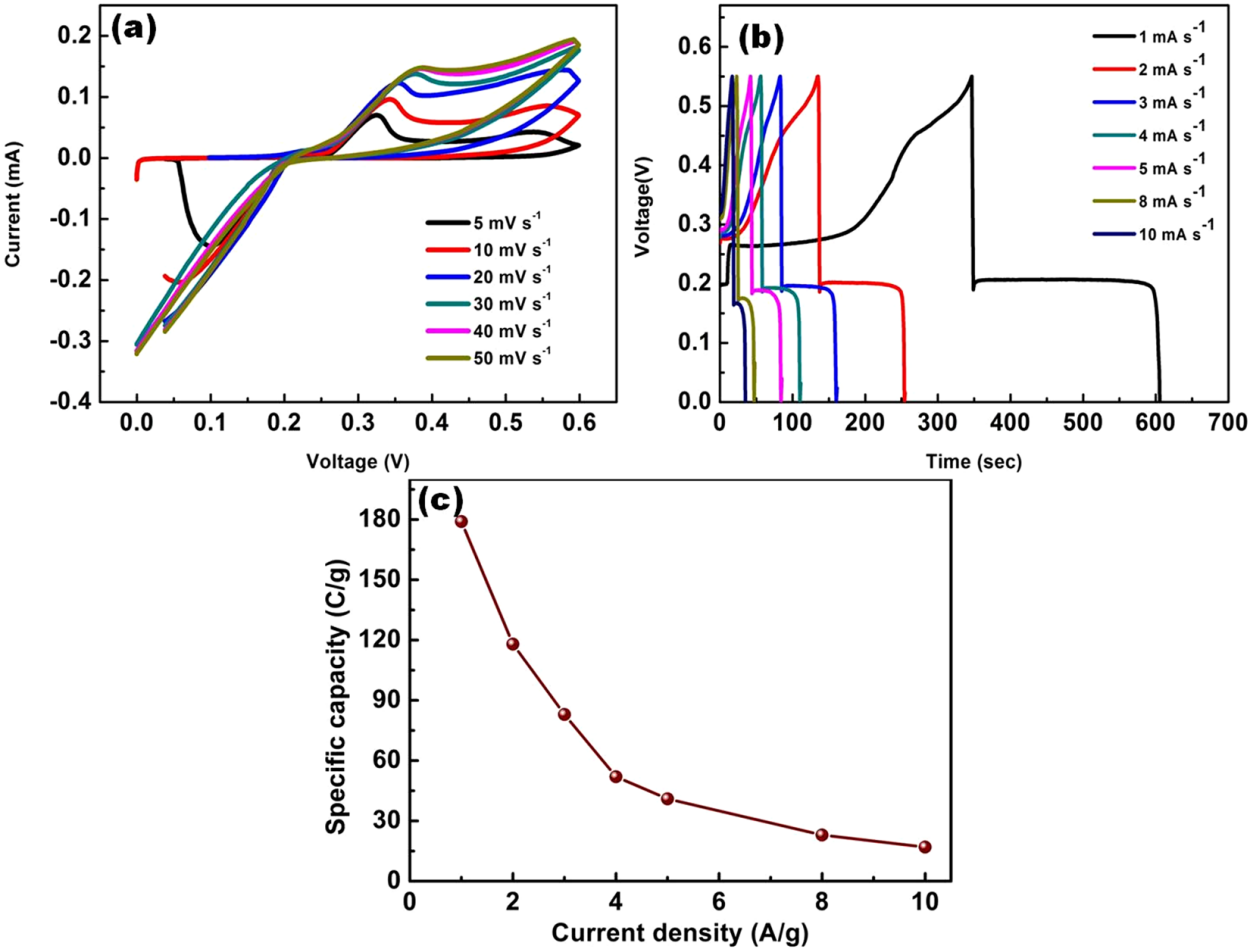
Electrochemical studies of Ag_2_S deposited on Ni mesh (**a**) CV profile, (**b**,**c**) CD and Specific capacity profile.

The recorded CV curve shapes discernible from the rectangular shape pointing out that the energy storage of Ag_2_S electrode impute to the faradaic capacity behaviour with two redox peaks^32,33^. The anodic and cathodic oxidation sweep lies at the range of 0.25–0.45 V and 0–0.2 V respectively (Fig. 5a). As the value of scan rate increases, there exists distortion in the symmetry of the CV curves and at the high scan rate an asymmetrical nature of the anodic and cathodic peak has been exists which may due to the kinetic irreversibility of the redox process^27^. The electrochemical behavior of Ag_2_S electrode in KOH electrolyte solution can be elucidated by following electrochemical reactions:

1 $${{\rm{Ag}}}_{{\rm{n}}}^{0}\iff {{\rm{Ag}}}_{{\rm{n}}}^{+}+{{\rm{e}}}^{-}$$

2 $${{\rm{Ag}}}_{2}{\rm{S}}+{{\rm{OH}}}^{-}\iff {{\rm{Ag}}}_{2}{\rm{S}}({\rm{OH}})+{{\rm{e}}}^{-}$$

Figure 5b shows the GCD profile of the Ag_2_S electrode measured at different current densities of 1–10 A/g with the potential window of 0–0.55 V. It clearly shows that there is voltage levelling-off period near 0.2 V in each discharge curve. Non-linear variation of Voltage Vs Time (charge/discharge), further entail a classic faradaic capacity behaviour resultant of occurrence of redox from the redox occurring at the electrode/electrolyte interfaces. The potential window between 0.2–0.5 V appeared to be non-symmetric, insulating battery behaviour showing IR drop. Despite the steep potential drops, prolonged plateau of output voltage is observed in the range of 0.2 V, which is due to the faradaic process that takes place in Ag_2_S electrode^34^. From the obtained GCD profile the specific capacitance can be calculated using the following equation^35^:

$${{\rm{C}}}_{{\rm{s}}}=\frac{I\ast {\rm{\Delta }}{t}}{m}$$

Where, C_s_ is the specific capacitance (F/g), *I* was the discharge current (A), m is the active mass of the material, $\triangle t$ is the discharge time (sec). C_s_ values are calculated from the GCD profile and the results were plotted in Fig. 5c. From Fig. 5c it is evident that the specific capacity drops with increasing current densities. C_s_ at different current densities of 1, 2, 3, 4, 5, 8 and 10 A/g are calculated and found to be 179, 118, 83, 52, 41, 23 and 17 C/g respectively. Ag_2_S electrode shows a maximum specific capacity of 179 C/g for 1 A/g current density, which seems to be higher compared to other reported sulphur based materials such as CuS (62 F/g), ZnS (32 F/g), WS_2_ (40 F/g), RuS_2_ (85 F/g)^36–39^. This sort of high specific capacitance can be allocated to its architecture providing rapid electron and ion transfer and easy access to electrolyte ions. The CV and GCD result confirms that the active material Ag_2_S are battery type electrode materials. The IR drop in GCD profile features the charge conduction and ion diffusion process. Even operating at higher current rate the charge curve and the discharge counterpart exist to symmetry indicating the good coulombic performance of the device^40^.

**Figure 6:**
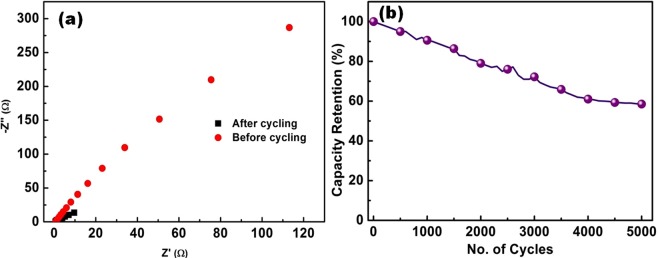
(**a**) EIS spectra and (**b**) Specific capacity retention.

The rate capability is a prime aspect of consideration in designing high power supercapacitors, which is evaluated from electrochemical impedance spectroscopy (EIS) studies^41,42^. Nyquist plot of Ag_2_S electrode, after and before cycling was carried out with frequency ranging from 100 kHz to 100 mHz as shown in Fig. 6a. An intercept with real axis at high frequency represents the series resistance, which is combination of ionic resistance of the electrolyte, electronic resistance of the electrode materials and interface resistance^43^. It is evident that there is no remarkable change in the external sheet resistance (ESR) after the cycling test, which indicates high ionic conductivity of the supercapacitors. A sharp increase of impedance towards lower frequency indicates the pure capacitive behaviour which arises from diffusion of redox species. The stability of the electrode materials plays a vital role for the practical applications of supercapacitors. Therefore, the cycling stability of the electrode was evaluated at 10 A/g for 5000 cycles as shown in Fig. 6b. The capacity retention of the active materials (Ag_2_S) maintains reasonable stability over the prolong period of 5000 cycles. It is evident from the data that Ag_2_S can serves as a remarkable electrode material in the development of high performance electrochemical behaviour owing to its excellent behaviour with good cyclability and high retention capacity.

### Microstructure analysis

**Figure 7:**
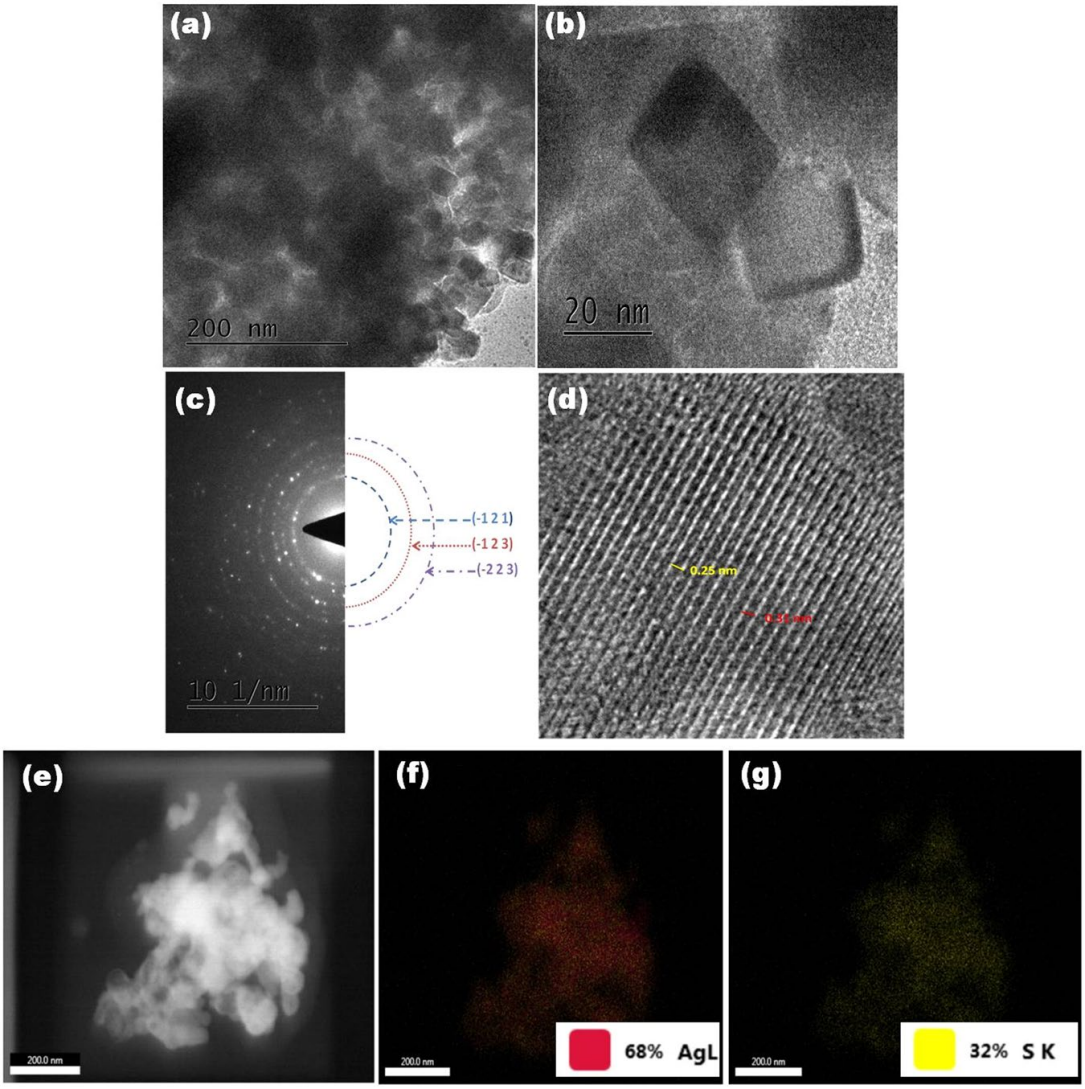
(**a**) HRTEM image, (**b**) higher magnification, (**c**) SAED pattern, (**d**) lattice fringes. (**e**) Elemental mapping of Ag_2_S, (**f**) Ag and (**g**) S.

The microstructure analysis of Ag_2_S material was carried out using HR-TEM analysis. The micrograph shown in Fig. 7a,b confirmed that as-synthesized Ag_2_S are smaller particles in the order of nanometer (nm) in range with the size of 20–25 nm. Figure 7c represents the SAED pattern of Ag_2_S nanoparticles, the observed ring profile was indexed and it corresponds to the plane of (−1 2 1), (−1 2 3), (−2 2 3). The high intensity spots observed in the inner ring matches 100% with the plane of (−1 2 1) confirming Ag_2_S nanoparticles are polycrystalline in nature. The lattice fringes of the Ag_2_S nanoparticles is clearly seen in Fig. 7d with the d-spacing of about 0.25 nm which closely matched to the standard value (0.260 nm) and indexed to the (−1 2 1) lattice plane. Figure 7e–g shows elemental mapping profile of Ag and S present in the sample. The mapping results show that Ag and S are uniformly distributed in the entire sample.

## Conclusion

In conclusion, we have reported the deposition of nanocubic Ag_2_S on the surface of Ni mesh by a simple and cost effective chemical bath deposition for the supercapacitor applications. The acanthite phase of Ag_2_S with good crystalline is confirmed from the XRD studies. The XPS studies emphasize the formation of Ag_2_S is in nearly stochiometric form. The electrochemical studies of Ag_2_S electrode show a considerable supercapacitance performance value of 179 C/g at 1 A/g. In addition, the Ag_2_S prepared by this method serves as an additive free electrode and exhibits its better performances in electrochemical studies. Thus, these results imply promising electrochemical behaviour of Ag_2_S electrode towards cutting edge applications in energy storage sectors.

## Methods

### Materials and characterization

Silver Nitrate (AgNO_3_, 98%, SRL, INDIA); Thiourea (CH_4_N_2_S, 98%, Merck KGaA, Germany); Ammonia Solution (NH_3,_ 25% GR, Merck, INDIA); Nickel Mesh (Ni) and Deionised water. All the chemicals and reagents used in this experiment are procured commercially with analytical grade and are used as such.

The phase structure and the crystalline behaviour of the fabricated Ag_2_S thin film was deliberated using X-Ray diffraction (XRD, Bruker, D8 Advance) with Cu K_α_ radiation (λ = 0.15406 nm) at a scanning rate of 0.05 s^−1^. Particle size of the sample was analyzed using High Resolution Transmission Electron Microscopy (HRTEM, Tecnai 20, G2, FEI) operating at 200 kV with capable of an information limit of 0.14 Å. The oxidation states of Ag and S in the Ag_2_S films were examined using X-ray Photoelectron Spectrometer (XPS, Thermo scientific model: MATLAB 2000), operating with an Mg source with hʋ = 1253.6 eV. Morphology of Ag_2_S deposited on Ni mesh was observed using Field Emission Scanning Electron Microscopy (FESEM, Carl Zeiss microscope, surpa-55VP). Prior to the analysis, surface of the samples were sputtered using gold (Au) for better electrical conductivity. The functional group present in the Ag_2_S sample was analysed using Fourier-transform Infrared Spectroscopy (FTIR, FTIR-6300 Japan, Model-Tensor 27).

Electrochemical characteristics of the binder-free Ag_2_S were evaluated using three electrodes configurations with 3.6 M KOH aqueous electrolyte. Ni mesh coated with Ag_2_S served as a working electrode, Platinum wire as counter electrode and Hg/HgO as reference electrode.

### Fabrication of binder-Free Ag_2_S electrodes

**Figure 8:**
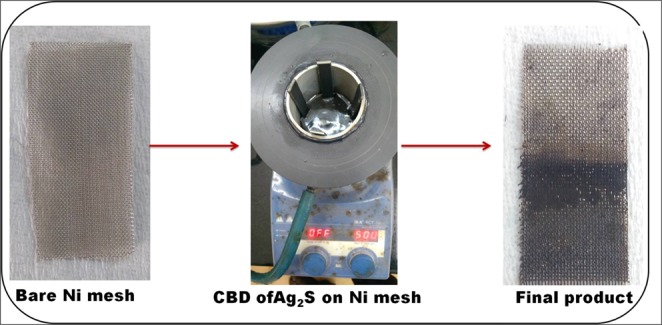
Pictorial representation of Ag_2_S deposition on Ni mesh via CBD.

In a typical fabrication of Ag_2_S on Ni mesh, an equimolar (0.1 M) ratio of silver nitrate and thiourea has been taken as source for silver and sulphur. Initially, the Ni mesh was placed on the bath which is maintaining at 6 °C temperature. Prior to addition of precursors Ni mesh undergoes treatment to remove native oxides as per earlier report^44^. Then the prepared silver nitrate solution was added on to the bath followed by addition of sulphur source (thiourea). To maintain the solution in basic nature (pH~9), few drops of ammonia solution was added to the homogeneous solution. An additional feature of this synthesis methodology was that it does not require any binders such as nafion or triton-X 100 for deposition of materials on the surface of Ni mesh electrode. After the experiment the loading of active materials on the Ni mesh was calculated by weighing the weight difference between before and after loading. The loading of active materials was found to be 0.6 mg. The schematic pictorial representation for CBD of Ag_2_S on Ni mesh is shown in Fig. 8.

## Data Availability

Readers can access the data via contact to the authors.
